# Polymorphisms in *K13, pfcrt, pfmdr1, pfdhfr*, and *pfdhps* in parasites isolated from symptomatic malaria patients in Burkina Faso

**DOI:** 10.1051/parasite/2016069

**Published:** 2016-12-22

**Authors:** Anyirékun Fabrice Somé, Hermann Sorgho, Issaka Zongo, Thomas Bazié, Frédéric Nikiéma, Amadé Sawadogo, Moussa Zongo, Yves-Daniel Compaoré, Jean-Bosco Ouédraogo

**Affiliations:** 1 Institut de Recherche en Sciences de la Santé, Direction Régionale de l’Ouest 399 Avenue de la liberté 01 BP 545 Bobo-Dioulasso 01 Burkina Faso; 2 Institut de Recherche en Sciences de la Santé, Unité de Recherche Clinique de Nanoro BP 218 Ouaga CMS 11 Burkina Faso

**Keywords:** *Plasmodium falciparum*, *k13*, *pfcrt*, *pfmdr1*, *dhfr*, *dhps*, artemisinin resistance

## Abstract

*Background:* The emergence of resistance to artemisinin derivatives in western Cambodia is threatening to revert the recent advances made toward global malaria control and elimination. Known resistance-mediating polymorphisms in the *K13, pfcrt, pfmdr1, pfdhfr*, and *pfdhps* genes are of greatest importance for monitoring the spread of antimalarial drug resistance. *Methods*: Samples for the present study were collected from 244 patients with uncomplicated malaria in health centers of Bobo-Dioulasso, Burkina Faso. Blood sample was collected on filter paper before the subject received any treatment. The parasite DNA was then extracted and amplified by Polymerase Chain Reaction (PCR) to evaluate the prevalence of polymorphism of *pfcrt*K76T, *pfmdr1* (N86Y, Y184F), and *pfdhps* (A437G, K540E). The K13 gene polymorphism was analyzed by nested PCR followed by sequencing. *Results*: The overall results showed 2.26% (5/221) of K13 synonymous mutant alleles (two C469C, one Y493Y, one G496G, and one V589V), 24.78%, 19.58%, 68.75%, 60.9%, 53.7%, 63.8%, and 64.28%, respectively, for mutant *pfcrt 76T*, *pfmdr1-86Y, pfmdr1-184F, pfdhfr51I, pfdhfr59R, pfdhfr108N*, and *pfdhps 437G.* We did not report any mutation at codon 540 of *pfdhps*. *Conclusion*: These results provide baseline prevalence of known drug resistance polymorphisms and suggest that artemisinin combination therapies may retain good efficacy in the treatment of uncomplicated malaria in Burkina Faso.

## Abstract (fr)

Contexte : L’émergence de la résistance aux dérivés de l’artémisinine dans l’ouest du Cambodge menace d’annuler les avancées récentes en matière de lutte et d’élimination du paludisme à l’échelle mondiale. Les polymorphismes de médiation de résistance connus dans les gènes *K13, pfcrt, pfmdr1, pfdhfr* et *pfdhps* sont de la plus grande importance pour surveiller la propagation de la résistance aux antipaludiques. *Méthodes :* Les échantillons de cette étude ont été prélevés sur 244 patients atteints de paludisme non compliqué dans les centres de santé de Bobo-Dioulasso, au Burkina Faso. Les échantillons de sang ont été prélevés sur du papier filtre avant que le sujet ne reçoive aucun traitement. L’ADN du parasite a ensuite été extrait et amplifié par PCR pour évaluer la prévalence du polymorphisme de *pfcrtK76T*, *pfmdr1* (N86Y, Y184F) et *pfdhps* (A437G, K540E). Le polymorphisme du gène *K13* a été analysé par PCR imbriquée suivie d’un séquençage. *Résultats :* Les résultats globaux ont montré 2.26 % d’allèles mutants (5/221) pour *K13* (deux C469C, un Y493Y, un G496G et un V589V), et 24.78 %, 19.58 %, 68.75 %, 60.9 %, 53.7 %, 63.8 % et 64.28 %, respectivement, pour les mutants *pfcrt 76T, pfmdr1-86Y, pfmdr1-184F, pfdhfr51I, pfdhfr59R, pfdhfr108N* et *pfdhps* 437G. Nous n’avons signalé aucune mutation au codon 540 de *pfdhps*. *Conclusion :* Ces résultats montrent une prévalence très faible des polymorphismes de pharmacorésistance connus et suggèrent que les thérapies combinées à base d’artémisinine pourraient conserver une bonne efficacité dans le traitement du paludisme non compliqué au Burkina Faso.

## Background

In Burkina Faso, malaria is still the most important infectious disease with the highest rates of mortality and morbidity [17, 22]. Since 2005, the country has changed its first-line drug for malaria to artemether-lumefantrine (AL) and artesunate-amodiaquine (AS-AQ) following reported widespread resistance to chloroquine (CQ), but sulfadoxine-pyrimethamine (SP) is still recommended for chemoprophylaxis in pregnant women [13]. Several studies have since demonstrated the excellent efficacy of artemisinin-based combination therapies (ACTs) for the treatment of uncomplicated malaria in Burkina Faso [30, 37, 38]. However, the emergence of *Plasmodium falciparum* resistance to artemisinin derivatives in Cambodia [8] represents a serious threat to disease case management, and also to its global control and elimination. The spread of parasite resistance to antimalarial drugs from Asia to Africa has already occurred with CQ and SP [35], suggesting the need for reinforced surveillance of artemisinin resistance in Africa. Several mechanisms of resistance of *P. falciparum* to antimalarial drugs have been reported [14, 34]. For instance, resistance to CQ has been associated with mutations in the gene encoding *P. falciparum* resistance transporter (*pfcrt*) [5, 11] and a number of mutations in *P. falciparum* multidrug resistance 1 (*pfmdr1*) [2, 21]. The same polymorphic traits are also associated with resistance to amodiaquine (AQ) [6, 15] and with decreased susceptibility to lumefantrine [7, 24, 25]. In early 2014, artemisinin-resistant phenotypes in Cambodia were associated with mutation in *Pf3d7_1343700*, one exon gene, which codes for a putative Kelch protein 13 [1]. The identified mutations were associated *in vitro* with an increased survival rate and *in vivo* with delayed parasite clearance after artemisinin treatment. One year later, after the identification of artemisinin resistance-mediating mutations, another study clarified the role played by *K13* in artemisinin resistance. The authors found that when a K13 mutant parasite was repaired to wild type, the artemisinin resistance was lost; and that conversely, sensitive parasites become more resistant to artemisinin once the wild-type K13 gene is converted to mutant [27].

In West Africa, a study in Dakar, Senegal did not report any mutation between the six blades of K13 [33]. Another study investigating polymorphism in *K13 propeller* across 12 countries in sub-Saharan Africa identified more than 20 unique mutations. However, this study did not identify any of the mutations reported in south Asia [16].

Although ACTs have demonstrated high efficacy in the treatment of uncomplicated malaria in Bobo-Dioulasso, Burkina Faso, some reports have raised concerns linked to the existence of some selection of antimalarial drug resistance-mediating polymorphisms [25, 26]. To date, gene polymorphisms in *K13 propeller* have not been investigated in the Western region of the country, where malaria transmission is holoendemic. In this paper, we aimed to identify the baseline prevalence of known resistance-mediating mutations in the *K13 propeller, pfcrt, pfmdr1, pfdhfr*, and *pfdhps* genes in parasites isolated from symptomatic malaria patients in Bobo-Dioulasso, Burkina Faso.

## Material and methods

### Study area

The clinical study was conducted from October to December 2012 in the two peripheral health facilities of Colsama and Sakaby, situated approximately 15 km away from each other in Bobo-Dioulasso, Burkina Faso. Bobo-Dioulasso is located in the western region of the country where malaria transmission is holoendemic with a peak during the rainy season, approximately August to October, with an estimated entomological inoculation rate (EIR) of 300–500 infective bites per person per year.

### Patient and inclusion criteria

All patients aged 6 months or more and attending Colsama and Sakaby health centers with fever or history of fever in the last 24 h were referred by a clinician for the screening of malarial infection using Giemsa-stained thick and thin blood smears. Participants with *P. falciparum* infection at parasite densities between 2,000 and 200,000 parasites/μL and hemoglobin >5 g/dL were included in the clinical study after they or their parent/guardian signed an informed consent form. Other inclusion criteria were: absence of known adverse reactions to study drugs, absence of non-malarial febrile diseases, absence of documented malaria treatment in the last two weeks, and absence of warning signs or severe malaria.

### Sample collection and laboratory analysis

For all patients included in the study, thick and thin smears were collected and stained with 10% Giemsa on the day of inclusion and at any scheduled or unscheduled visit. Parasite densities were calculated by counting the number of asexual parasites per 200 leukocytes, assuming a leukocyte count of 8,000/μL of blood.

For Polymerase Chain Reaction (PCR) analysis, blood was also collected at the time of inclusion onto filter paper (Whatman 3MM, GE Healthcare), labeled, air-dried, and stored in sealed plastic bags at ambient temperature. Parasite DNA was subsequently extracted using the QIAamp DNA Mini Kit (Qiagen, Germany) according to the Qiagen-DNA purification from dried blood spot protocol [20].

The propeller domain (codons 440–680, 720 bp) of the *K13* gene was amplified by nested PCR using the protocol previously described [1]. For quality control, six *P. falciparum* strains with known *K13* alleles were extracted and analyzed at the same time as study samples. Aliquots of PCR products were analyzed by gel electrophoresis to confirm amplification before sending the remaining PCR products for sequencing at the Wellcome Trust Sanger Institute, Hinxton, United Kingdom. PCR products were sequenced by Macrogen and electropherograms analyzed on both strands using PF3D7_1343700 as the reference sequence.

Single nucleotide polymorphisms (SNPs) in the *pfcrt* gene at codon 76, *pfmdr1* at codons 86 and 184, *pfdhps* at codons 437, 540, and *pfdhfr* at codons 51, 59, 108, and 164 were assessed using nested PCR, followed by restriction enzyme digestion, as previously described [9, 10]. Digestion products were resolved by gel electrophoresis, and the results were classified as wild type, mixed, or mutant, based on the migration patterns. Samples containing both wild-type and mutant alleles were classified as mutant.

### Statistical analysis

Data were collected with EpiData and analyzed by STATA version 12 (STATA, CA, USA). The count of samples with wild-type and mutant alleles was used to generate the prevalence of the alleles. The chi-square test was used to compare proportions and the statistical significance was defined as a *p*-value, 0.05.

### Ethics

All the study participants provided informed consent before their enrollment and the study was approved by the institutional Ethics Committee of Centre Muraz, Burkina Faso. All participants received adequate antimalarial treatment.

## Results

A total of 244 samples were collected from patients with uncomplicated *P*. *falciparum* malaria in two primary health centers of Bobo-Dioulasso. The study population was composed of 111 males and 133 females with a mean age of 26.91 ± 18.11 years. The minimum and maximum ages recorded were, respectively, 0.6 and 60 years. The mean body axillary temperature measured prior to blood sampling was 38.51 ± 1.18 °C and the mean parasite count was 52,034 ± 47,590 trophozoites per μL of blood.

### Prevalence of mutation in the *K13 propeller*

The gene encoding the propeller domain was successfully sequenced in 221/244 malaria episodes diagnosed from July to December 2012 (Table 1). Using pf3D7_1343700 as the reference strain, we reported 2.26% (5/221) of samples harboring the *K13* synonymous mutant alleles (two C469C, one Y493Y, one G496G, and one V589V). SNPs that were associated with *in vitro* resistance or delayed parasite clearance in Southeast Asia were not observed in Bobo-Dioulasso, nor were any of the polymorphisms observed in parasites from Southeast Asia, nor the M476I mutation that was selected *in vitro* with artemisinin pressure.

**Table 1.** PCR sensitivity for mutation points analyzed in different *Plasmodium falciparum* genes.

	*K13*	*Pfcrt k76T*	*Pfmdr1 (Y184F, N86Y)*	*Pfdhfr (N51I, S108N)*	*Pfdhfr C59R*	*Pfdhps (A437G, K540E)*
Number of samples	228	244	244	244	244	244
Genotyping success *n* (%)	221 (96.9)	236 (96.7)	238 (97.5)	243 (99.6)	242 (99.2)	237 (97.1)

### Prevalence of *pfdhfr* and *pfdhps*

**Figure 1.**
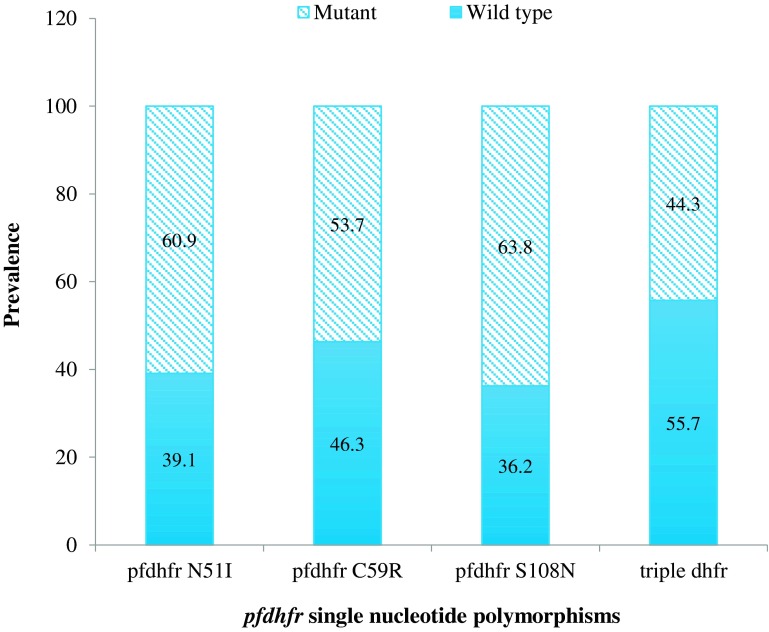
Prevalence of pfdhfr and pfdhps polymorphisms.

The prevalence of *pfdhfr* 51I, 59R, 108N, and *pfdhps* 437G mutations was reported, respectively, in 60.9% (148/243), 53.7% (130/242), 63.8% (150/235), and 64.0% (151/236) of the samples. We did not find any mutation at codons 164 of *pfdhfr* and 540 of *pfdhps*. Triple pure mutant *pfdhfr* (51, 59, and 108) genes were found in 90 samples and mixed triple mutants in 14 samples (Fig. 1).

### Prevalence of *pfcrt* and *pfmdr1*

Among 236 samples successfully amplified for *pfcrt*, a mutation was found at codon K76T in 58/236 (24.6%) samples. We did not evaluate the *pfcrt* 72–76 haplotypes in these samples.

**Figure 2.**
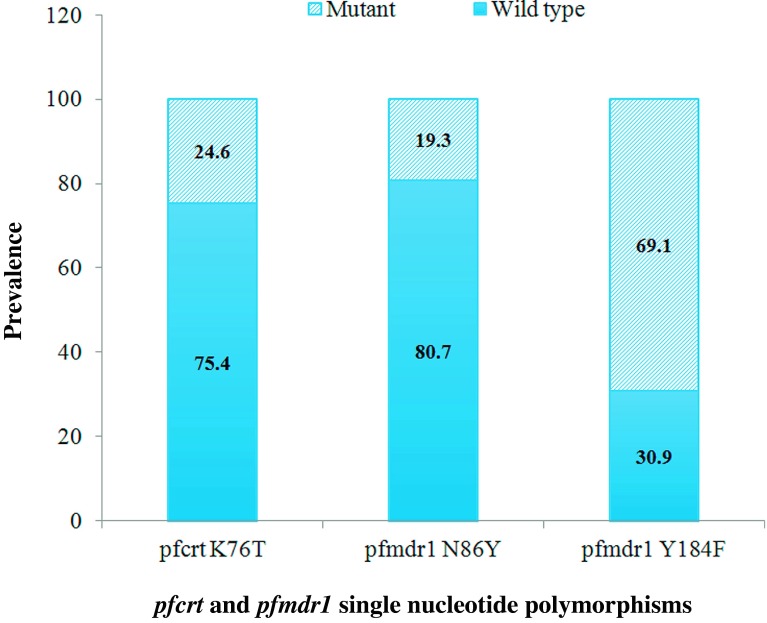
Prevalence of *pfcrt* and *pfmdr1* polymorphisms.

Mutations in *Pfmdr1 N86Y* and *Y184F* were identified in 46/238 (19.3%) and 167/236 70.8% samples, respectively (Fig. 2). We did not genotype three additional mutations in *pfmdr1* (S1034, N1042, and 1246) that are important elsewhere. These mutations are reported to be uncommon or absent in Burkina Faso throughout the literature.

## Discussion

Despite significant control efforts, malaria remains a major concern in Burkina Faso, with 63.2% of hospitalizations and 49.6% of deaths among children under 5 years of age in the country [23] (PNLP, 2014). Our study aimed to evaluate the baseline prevalence of known resistance-mediating mutations in the *k13 propeller*, *pfcrt*, *pfmdr1*, *pfdhfr*, and *pfdhps* genes in parasites isolated from symptomatic malaria patients in Bobo-Dioulasso, Burkina Faso. The study is justified by the need for surveillance of *P*. *falciparum* resistance to antimalarial drugs, in contribution to the monitoring of ACT efficacy in Burkina Faso.

Analysis of *K13 propeller* did not detect any of the polymorphisms associated with artemisinin resistance in Southeast Asia [1]. Overall, we showed very low prevalence of *k13 propeller* synonymous mutations. This result is in line with recent studies, which were not able to find any of the polymorphisms associated with artemisinin resistance in sub-Saharan Africa [16, 28]. The absence of known mutations in the *k13 propeller* gene augurs favorably for the antimalarial efficacy of ACTs at least in the western part of Burkina Faso with high malaria transmission. Nevertheless, a recent study reported the identification of a unique *K13* mutation from Ethiopia [4]. This R622I mutation is located on blade 5 of the *K13 propeller* domain and had not been seen in previous investigations in Africa. More careful monitoring of the evolution of this resistance marker is needed in malaria endemic regions of Africa in order to anticipate countermeasures. In our study, high numbers of undetermined K13 polymorphisms have been reported because PCR and/or sequencing failures could not be repeated due to limited funding. This constituted one of the limitations of the study.

Concerning polymorphisms in *pfdhfr* and *pfdhps*, which mediate resistance to antifolates, four well-characterized mutations in *pfdhfr* (51I, 59R, 108N) and *pfdhps* (437G) were very common in Bobo-Dioulasso at high prevalence. The presence of mutations at codons 540 of *pfdhps* and 164 of *pfdhfr* was not detected in our sample, consistent with previous studies in the subregion [20, 32]. In Burkina Faso, SP is used as monotherapy for intermittent preventive treatment in pregnancy (IPTp) and in association with amodiaquine for seasonal malaria chemoprevention (SMC), which is now being largely implemented in the Sahelian countries. Despite the absence of *pfdhps* 540E, the high prevalence of mutations reported in *pfdhfr* (51I, 59R, and 108N) and *pfdhps* 437G may jeopardize the efficacy of SP as a tool for malaria control in Burkina Faso. It is therefore very important to continuously monitor the prevalence trend of *pfdhfr* and *pfdhps* mutations for their direct impact on the efficacy of IPTp programs.

For the *pfcrt 76T* mutation, which is the main determinant of CQ resistance [5], and which is also associated with AQ resistance, we reported a significant decrease of its prevalence as compared to previous studies in Burkina Faso [6, 25, 26, 31]. A similar dramatic decrease in the prevalence of the *pfcrt 76T* mutation has been reported in Malawi following the withdrawal of CQ for the treatment of malaria [12, 18]. This “chemo-reversion” is interpreted as the result of rapid re-expansion of susceptible parasite strains that survived in semi-immune hosts during the periods of high chloroquine drug pressure [19], and this phenomenon might be accelerated in areas where lumefantrine is currently used [29].

The *pfmdr1* mutation encodes a predicted food vacuole transporter homologous to P glycoproteins, which mediate resistance in cancer cells by increasing the efflux of chemotherapeutic agents. For *pfmdr1 86Y*, the prevalence of wild-type alleles decreased over time in comparison to previous studies [25, 26]. This result is consistent with recent findings [3] and is likely due to the withdrawal of CQ and the widespread use of AL as first-line antimalarial treatment in the country, which promoted the selection of the wild-type sequences at these alleles [6, 25, 26]. In Burkina Faso, *pfmdr1 86Y* and AQ resistance has been investigated. The study indicated that *pfmdr1* could be useful in monitoring AQ resistance [31]. Mutant *pfmdr1*-*184F* was most prevalent in our sample but this mutation is not associated with susceptibility to various antimalarial drugs [36]. Although only minor associations with drug susceptibility have previously been observed with polymorphisms at the *pfmdr1 184F* locus, one previous study suggests that this allele may also play a role in mediating resistance to some antimalarials [7].

## Conclusion

This study did not find any of the known *K13* mutations associated with artemisinin resistance. However, the study reported high prevalence of mutations in *pfdhfr* and *pfdhps* associated with SP resistance. Thus, the use of known molecular markers is fundamental for surveillance in malaria control programs in order to prolong the life span of artemisinin-based combination therapy (ACT) in Africa.
